# Safety Evaluation of a Bioglass–Polylactic Acid Composite Scaffold Seeded with Progenitor Cells in a Rat Skull Critical-Size Bone Defect

**DOI:** 10.1371/journal.pone.0087642

**Published:** 2014-02-03

**Authors:** Karam Eldesoqi, Dirk Henrich, Abeer M. El-Kady, Mahmoud S. Arbid, Bothaina M. Abd El-Hady, Ingo Marzi, Caroline Seebach

**Affiliations:** 1 Department of Trauma-, Hand- and Reconstructive Surgery, Hospital of the Goethe- University, Frankfurt/Main, Germany; 2 Department of Biomaterial, National Research Centre, Cairo, Egypt; 3 Department of Pharmacology, National Research Centre, Cairo, Egypt; Université de Technologie de Compiègne, France

## Abstract

Treating large bone defects represents a major challenge in traumatic and orthopedic surgery. Bone tissue engineering provides a promising therapeutic option to improve the local bone healing response. In the present study tissue biocompatibility, systemic toxicity and tumorigenicity of a newly developed composite material consisting of polylactic acid (PLA) and 20% or 40% bioglass (BG20 and BG40), respectively, were analyzed. These materials were seeded with mesenchymal stem cells (MSC) and endothelial progenitor cells (EPC) and tested in a rat calvarial critical size defect model for 3 months and compared to a scaffold consisting only of PLA. Serum was analyzed for organ damage markers such as GOT and creatinine. Leukocyte count, temperature and free radical indicators were measured to determine the degree of systemic inflammation. Possible tumor occurrence was assessed macroscopically and histologically in slides of liver, kidney and spleen. Furthermore, the concentrations of serum malondialdehyde (MDA) and sodium oxide dismutase (SOD) were assessed as indicators of tumor progression. Qualitative tissue response towards the implants and new bone mass formation was histologically investigated. BG20 and BG40, with or without progenitor cells, did not cause organ damage, long-term systemic inflammatory reactions or tumor formation. BG20 and BG40 supported bone formation, which was further enhanced in the presence of EPCs and MSCs.

This investigation reflects good biocompatibility of the biomaterials BG20 and BG40 and provides evidence that additionally seeding EPCs and MSCs onto the scaffold does not induce tumor formation.

## Introduction

Large bone defects are still a major challenge in orthopaedic and trauma patients. Bone is a dynamic, tightly vascularized tissue with an anrivaled capacity for regeneration [1]. But if the defect exceeds a critical size, endogenous rengeneration processes fail to bridge the gap [1]–[3]. At present, such critical size bone defects are best treated using autologous bone grafts harvested from the patient's own iliac crest. However, the limited amounts of bone available for grafting, elevated costs resulting from multiple procedures and associated complications such as donor site morbidity, are limiting factors, which promote the search for new approaches to optimize fracture-healing [4].

Tissue engineering can potentially provide treatment alternatives for conventional large bone defects [5]. The application of different combinations of osteoconductive biomaterials, osteoprogenitor cells and growth factors, directly into the defect, holds great potential for achieving optimal bone healing under difficult circumstances [5].

Biomaterials serve as matrices for tissue formation, and thus should ideally fill multiple roles including mechanical strength, biodegradability and support and differentiation of regenerative cells [6]. To date, no biomaterial fulfils all these criteria. Combining biomaterials with some desirable characteristics may circumvent these limitations. Hence, several studies have focused on developing and characterizing biodegradable and bioactive porous polymer/inorganic composite biomaterials. The composite materials are designed to mimic bone-forming components to elicit specific cellular responses and provide an ideal environment for bone formation [6], [7]. In the present study a composite material based on bioglass (BG) and poly-L-lactic acid (PLA) is introduced.

Bioglass, consisting of calcium oxide and silicate (CaO-SiO_2_), has been shown to stimulate the formation, precipitation and deposition of calcium phosphates from physiological solution and can result in enhanced bone-matrix interface strength [8], [9]. The biocompatibility of silicate based glass has long been established [10], [11]. When implanted in a biological system BG undergoes chemical degradation, releasing ions (Na^+^, Ca^2+^) and converts to an hydroxy-apatite material. Silicon is also released during the degradation process and is presumably harmlessly secreted in a soluble urinary form [12].

Polylactic acid (PLA) is a polymer of lactic acid with high biocompatibility. In the living body PLA hydrolizes to its constituent α-hydroxy acid, which is then incorporated into the tricarboxylic acid cycle and excreted [13], [14]. The degradation products of PLA are not toxic [15] and PLA has been approved by the Food and Drug Administration (FDA) for biomedical application [16].

Composite biomaterials made of a polymer and bioglass have been established in the past few years [9]. Composite biomaterials take advantage of the osteoinductive properties of bioglass and the strengthening effect of the polymeric component [17]. Several combinations were under investigation. For example Xu et al demonstrated the excellent biocompatibility and bone regenerative capacities of a biomimetic scaffold consisting of bioglass, collagen and phosphatidylserine [18], [19]. Composite biomaterials based of PLA and bioglass had been also developed and demonstrated a good eligibility for cell based tissue engineering approaches [17], [20]–[23].

Reparative cells are also an important aspect in bone tissue engineering and they require a biomaterial scaffold, which positively influences cell adhesion, morphology, proliferation and differentiation of neighbouring cells [24]. Scaffolds seeded with mesenchymal stem cells (MSC) have been used to treat critical size defects (CSD) in human and animal models [25]–[27]. Furthermore, it has been demonstrated that osteogenic prestimulation of MSC results in significant improvement in the bone healing response [28], [29]. Depending on the size of the bone defect, the in-growth of bone-forming cells may be limited due to lacking vascularization and insufficient nutritional bone graft support. Thus, early vascularization of the composite material in the bone defect is a crucial step for in-growth of osteogenic reparative cells in regenerating bone *in vivo*. Improved early vascularization has been achieved by using endothelial progenitor cells (EPC) [30]–[32].

Although there is broad evidence that neither bioglass nor PLA is toxic, adequate testing is mandatory for polymers to be used in biological systems. Before the use in humans it must be proved that the material is biocompatible and not cytotoxic. I*n vitro* studies alone are not suitable for assessing biocompatibility since the implantation site situation can hardly be mimicked. Processes such as clearing of degradation products, local interaction with different cell populations and inflammatory reactions can only be accurately investigated *in vivo*. Adequate testing assesses acute and systemic toxicity, carcinogenicity, encapsulation of the implant, hemolysis and pyrogenicity [33].

Besides testing the new material, it is important to determine whether the transplanted cells are tolerated or if adverse reactions or tumour formation are induced. It has been shown that *in vitro* expansion of MSC can lead to chromosomal aberration [34]. Also, some of the growth factors that are used for EPC differentiation *in vitro* such as IGF-1 might support transformation of hematopoietic progenitors [35], from which EPCs develop [36].

Hence, the aim of the present study was to carry out a comprehensive safety evaluation of a newly developed composite material consisting of PLA and 20 or 40% bioglass (BG20 and BG40, respectively), seeded with EPCs and differentially pretreated MSCs. Custom fit scaffolds with or without prior cell loading were implanted into critical size skull defects in rats. Over a period of 3 months the implantation site was histologically monitored for inflammation. Blood biochemical parameters were monitored by means of clinical chemistry and liver, kidney and spleen were histologically monitored for tumor occurrence.

## Materials and Methods

### Ethics

All animal experiments were approved and performed in accordance with regulations set forth by our institution's animal care and oversight committee, located at the Regierungspräsidium Darmstadt (Regierungspräsidium Darmstadt – Veterinärdezernat - Tierschutzkommission, Darmstadt, Germany, Project No. F3/22), in accordance with German law. All surgery was performed under general anesthesia, administered intraperitoneally as a mixture of Ketavet and Rompun. All efforts were made to minimize suffering. The animals were sacrificed with an overdose of intraperitoneal pentobarbital (150 mg/kg).

### Fabrication of the biomaterials: PLA, BG20 and BG40

**Figure 1:**
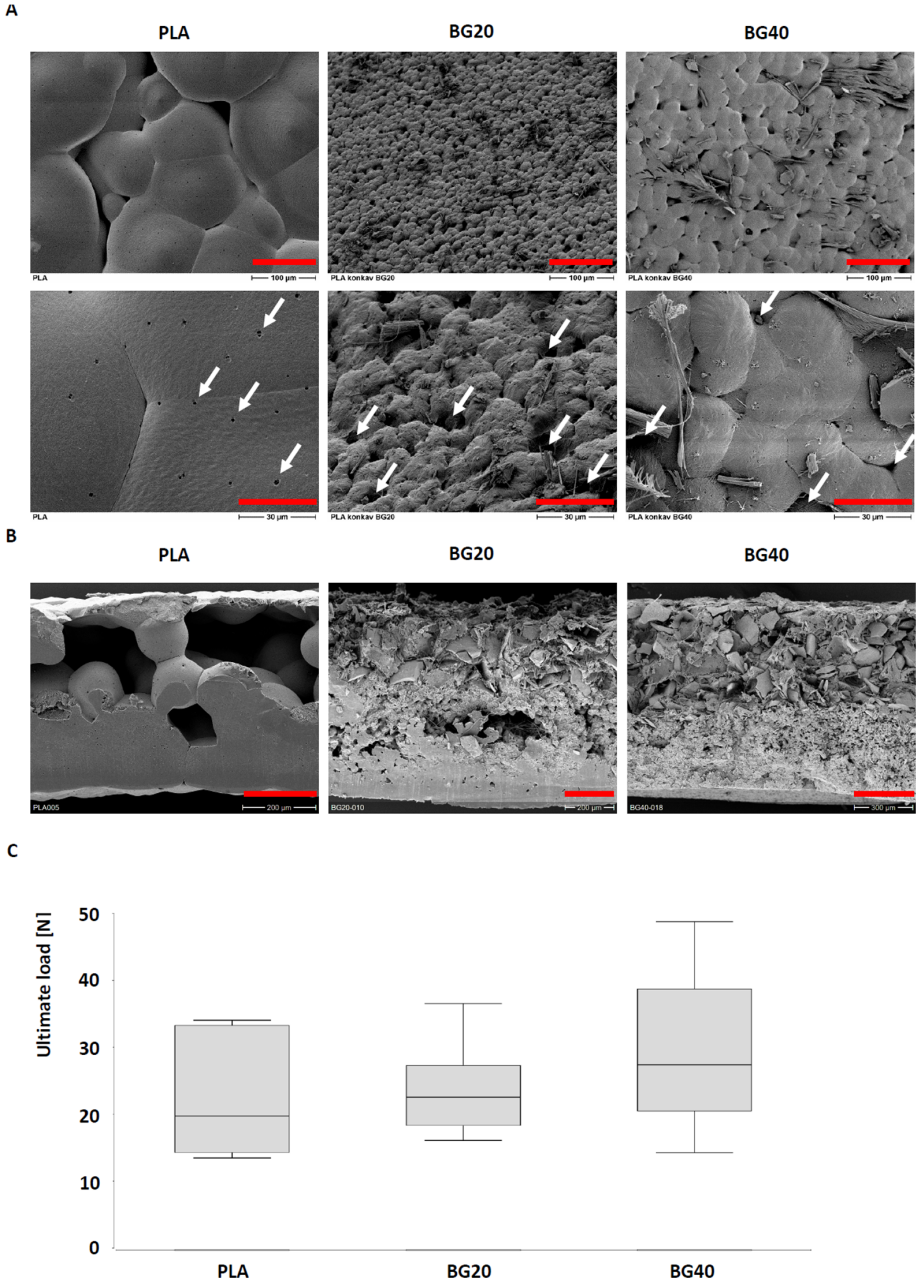
Surface characteristics and and biomechanical properties of disc shaped PLA, BG20 and BG40. Surface characteristics were analysed by SEM (A). The upper row provides an overview, the lower row presents a detailed view on distinct surface characteristics. Biomaterials with bioglass demonstrated a much more jagged surface with greater pores (arrows) than pure PLA. Red scale bar: 100 µm (upper row), 30 µm (Lower row). A sagittal cross section of PLA, BG20 and BG40 is shown in (B). PLA (left) appeared relatively homogenous with great cavities and pores, whereas BG20 (middle) and BG40 (right) demonstrated a biphasic structure. The upper part of BG20 and BG40 is characterized by an amorphic structure with pores and channels whereas the lower part has a higher density with few small pores. Red scale bar: 200 µm (PLA, BG20), 300 µm (BG40). The ultimate load of the disc shaped biomaterial specimen is depicted in (C). The median ultimate load as measured by a three point bending test increases slightly with the bioglass content of the biomaterials (n = 8, not significant).

The composite biomaterials consist of a PLA-component supplemented with two different amounts of bioglass. Tetraethyl orthosilicate (TEOS, ≥99%) and nitric acid 65% were supplied by Merck Chemicals KgaA (Darmstadt, Germany). Calcium nitrate [Ca (NO_3_)_2__4H_2_O, ≥99%], Poly (L-lactide) and chloroform (CHCl_3_, ≥99.4%) were purchased from Sigma-Aldrich (Steinheim, Germany). All chemicals were reagent grade and used as received without further purification. For the synthesis of bioglass CaO-SiO_2_ (SiO_2_ 80 mol-%, CaO 20 mol-%), a low viscosity gel was obtained by mixing 31 mL of tetraethyl orthosilicate (TEOS) and 8.6 g of Ca(NO_3_)_2_.4H_2_O in a solution of 5.5 mL of HNO_3_ 2M, used as catalyst, in 31.5 mL of H_2_O. The initial pH was 0.5. The bioglass was cast at room temperature in a Teflon container (Thermo Scientific Nalgene, Germany) until the gel was formed. Aging took place at 60°C for 3 days. Drying was carried out at 120°C. The glass was collected in a laboratory porcelain crucible (Haldenwanger GmbH, Waldkraiburg, Germany) and then progressively heated in a muffle furnace (Nabertherm GmbH, Lilienthal, Germany) at a rate of 3°C/minute to 700 C° and held for 3 hours. The glass particles were ground in a small porcelain mortar (Haldenwanger GmbH, Waldkraiburg, Germany) to form glass powder. Finally, the bioglass particles were sieved to be in the range of 106 µm–125 µm by sieves with a mesh size of 106 µm and 125 µm (Retsch GmbH, Haan, Germany). Composite biomaterials were prepared by mixing polymer [poly(L-lactide)(PLA)] and bioglass (BG) with 10 ml chloroform as follows: PLA, PLA/BG 20% and PLA/BG 40% biomaterials. The bioglass content was 0, 20 and 40%wt, respectively. These biomaterials are referred to as PLA, BG20 and BG40. Disc shaped specimens with a diameter of 5 mm and a thickness of 1 mm were cut and stored at room temperature under sterile conditions until use (Fig. 1a).

### Scanning electron microscopy

Adhesion of EPCs and MSCs to the biomaterials was assessed by scanning electron microscopy (SEM). Biomaterials seeded with cells were fixed 1 h after cell seeding with glutardialdehyde for 30 min and subsequently dehydrated by 15 min incubation in a 5-step ethanol gradient (25%, 50%, 75%, 96%, 100% ethanol). Biomaterials were then incubated overnight in 1,1,1,3,3,3-hexamethyldisilazane (Merck-Schuchardt, Hohenbrunn, Germany) and drained. To analyse the internal structures of the biomaterials 2 mm stripes including the center regions, were gently cut out of the disc shaped specimen using a scalpell. The samples were sputtered with gold (5×60 s, Agar Sputter Coater, Agar Scientific Ltd., UK) using a Hitachi FE-SEM S4500 (Hitachi, Dusseldorf, Germany) with a voltage of 5 kV. Images were digitally recorded using the Digital Image Processing System 2.6 (Point Electronic, Halle, Germany).

### Biomechanical properties of the biomaterials

The mechanical properties of the biomaterial discs (5 mm diameter) were evaluated using a destructive 3 point bending test. Individual discs consisting of PLA, BG20 respectively BG40 were placed on two pointed bars (distance 2.0 mm) on a mechanical test machine (*Zwickiline Z5.0*, Zwick-Roell, Ulm, Germany). The bending until failure was performed with a single pointed bar which was lowered with a constant speed of 0.1 mm/s. Load and deflection were recorded continuously by transducers coupled to measuring bridges and the ultimate load was calculated using the software *Testexpert II* (Zwick-Roell). The experiment was performed with each 8 individual samples of PLA, BG20 and BG40.

### Isolation of EPCs from rat spleen

Rat EPCs were isolated from the spleen of homozygous male Sprague-Dawley rats. The spleen was cut in small pieces (approximately 3 mm) and subsequently gently mechanically mashed, using a syringe plunger. The cell suspension was filtered through a 100 µm cell strainer (BD Biosciences), washed once with PBS and subjected to density gradient centrifugation (30 min, 900 g) with Biocoll (1.077 g/ml, Biochrom, Berlin, Germany). Cells were washed twice with cold PBS w/o (10 min, 900 g) and 2×10^6^ cells/cm^2^ were cultivated on fibronectin coated (10 µg/ml, Sigma, Deisenhofen, Germany) culture dishes with endothelial basal medium (EBM, Cambrex, Verviers, Belgium) supplemented with EGM Single quots at 37°C, 5% CO_2_. After 48 hrs non- and weakly-adherent cells were removed, the medium was changed and the cells were cultivated for an additional 72 hrs. A parallel preparation was performed to evaluate the percentage of endothelial differentiated cells. EPCs were identified by staining with 1,1′-dioctadecyl-3,3,3′,3′-tetramethylindo-carbocyanine-labeled acetylated low density lipoprotein (DiLDL, Cell-Systems, St. Katharinen, Germany) in EBM supplemented with 20% FCS. Only preparations with endothelial like differentiated cells greater than 80% were used. For the experiments the cells were detached by accutase treatment (10 min) (PAA-laboratories, Linz, Austria), washed once with EBM + supplements (Cell-Systems, St. Katharinen, Germany) and subsequently adjusted to a density of 5×10^5^ cells in 50 µl.

### Isolation and culture of MSCs from rat femur

Femurs were removed and cleaned. The condyles were cut using a site cutter and the bone marrow was flushed with a sterile syringe filled with PBS supplemented with 1% penicillin and streptomycin (P/S). The cells were recovered and washed once with PBS. The cell pellet was re-suspended in DMEM supplemented with 10% FCS and 1% P/S and directly transferred to a 75 cm^2^ culture flask. One culture flask per femur was used. Medium was exchanged twice a week. Cell passaging was performed when 80% confluency was reached. Cells were detached by 10 min incubation with accutase (PAA-laboratories), then washed (10 min, 300 g), re-suspended in PBS and adjusted to a density of 5×10^5^ cells in 50 µl. The cells used for the experiments were in the 3^rd^–5^th^ passage.

### Osteogenic differentiation of rat mesenchymal stem cells

MSCs were transferred to a new 75 cm^2^ culture flask and incubated with DMEM supplemented with 10% FCS, dexamethasone [1 µM], ascorbic acid [50 µg/ml] and β-glycerol phosphate [0.1 M] for three weeks. The medium was exchanged twice a week. A parallel setup was used to confirm the osteogenic differentiation by von Kossa staining. Osteogenic substances were purchased from Stem Cell Technologies, Grenoble, France).

### Cell seeding

2.5×10^5^ EPC and 2.5×10^5^ MSC osteogenic differentiated MSC (dMSC) in a volume of 10 µl PBS were carefully layered onto scaffolds and incubated for 1 h at 37°C.

### Animals and cell transplantation

#### Animal care

Eight-week old male Sprague-Dawley rats (n = 74, Charles River, Germany), weighing approximately 350–400 g were housed, four animals per cage, in temperature (15–21°C), air flow and light (12 h day & 12 h night) controlled rooms and received rat food and water ad libitum. The rats were randomly allocated to the experimental groups. Animals in the control group received a critical size skull defect (CSD), but no scaffold was implanted. The other groups received implants seeded with EPCs and differentially pretreated MSCs as shown in Table 1.

**Table 1:** Group setup and number of animals per group.

Group	Designation	Scaffold	cells	Animals, n =
**1**	empty	-----	-----	6
**2**	PLA	PLA	-----	6
**3**	BG20	PLA+20%BG	-----	7
**4**	BG40	PLA+40%BG	-----	7
**5**	PLA+MSC	PLA	MSC, EPC	8
**6**	BG20+MSC	PLA+20%BG	MSC, EPC	8
**7**	BG40+MSC	PLA+40%BG	MSC,EPC	8
**8**	PLA+dMSC	PLA	Ost. diff. MSC, EPC	8
**9**	BG20+dMSC	PLA+20%BG	Ost. diff. MSC, EPC	8
**10**	BG40+dMSC	PLA+40%BG	Ost. diff. MSC, EPC	8

PLA = polylactic acid. BG20 = PLA + 20% bioglass, BG40 = PLA + 40% bioglass. MSC = mesenchymal stem cells, dMSC = osteogenic predifferentiated MSC, EPC = endothelial progenitor cells.

General anesthesia (mixture of Ketavet and Rompun) was given intraperitoneally. To create a CSD in the skull, the head was shaved and cleaned with antiseptic fluid. A lateral longitudinal incision over the head was made under aseptic conditions. The skull cortex was drilled (X CUBE V2.0 drill, Avtec Dental, USA) using a 6 mm Trephine bur (VWR International GmbH, Darmstadt,Germany), so that a circular critical calvarial bone defect of 6 mm was created. The biomaterials were implanted into the defect zone and their position was checked. The wound was then closed with continuous subcutaneous stiches using a 4/0-monofilament nylon suture. Animals had free access to food and water and were monitored daily in the postoperative period for any complications or abnormal behaviour.

### Blood sampling, skull and internal organ collection

After 3 months the animals were sacrificed with an overdose of pentobarbital (150 mg/kg intraperitoneally) and weighed. Blood was collected from the abdominal aorta in blood collection tubes for serum and plasma for hematological tests (EDTA- and serum monovettes, Sarstedt,Nümbrecht,Germany). Liver, kidney and spleen were removed, weighed, fixed in 4% zinc-formalin (Thermo Scientific, Schwerte, Germany) and embedded in paraffin. The skull bone was dissected free and wrapped in gauze humidified with physiologic NaCl-solution and stored at −80°C until preparation for immunhistological examination.

### Histology

Paraffin embedded organ sections (5 µm) were stained with haematoxilin and eosin (HE).

Skull bones were decalcified over 7 days in a 10% Tris buffered EDTA-solution under continuous stirring and embedded in paraffin. Sections (5 µm) of the decalcified specimens were taken parallel to the long axis of the head and stained with HE. One slide per animal was analyzed by an independent observer blinded to group assignation using light microscopy (Axioobserver Z1, Zeiss) at a magnification of 100× in combination with a computer-supported imaging picture analysis system (Axiovision 4.7; Zeiss).

### Haematological analysis

The haemoglobin concentration as well as the number of red and white blood cells per µL were measured by conventional blood analysis (Hemavet instrument, DiaSys Greiner, Flacht, Germany).

### Serum biochemistry

GOT, GPT and ALP, as well as concentrations of creatinine, urea, glucose and total protein were measured using the “Spotchem SP-4430” analyzer (Medizintechnik Frank Guder GmbH, Bad Oeynhausen, Germany). Serum concentrations of NO_2_, malondialdehyde (MDA), glutathione (GSH) and superoxide dismutase (SOD) activity were measured by colorimetric assay kits (Cayman Biomol GmbH, Hamburg, Germany) and evaluated using Magellan v 6.5 software (Austria GmbH Grodig, Austria).

### Statistics

Results are presented as median (25% quartile/75% quartile), non parametric Kruskal-Wallis testing was applied followed by a Bonferroni-Holm adjusted multiple Conover-Iman posthoc analysis. A p value<0.05 indicates significance. Statistics were calculated using the software Bias 10.03 (Epsilon-Verlag, Darmstadt, Germany).

## Results

### Characterization of Biomaterials

The SEM analysis demonstrated a smooth a relatively dense surface of the PLA scaffold. The surface is characterized by relatively large alveolar and undulating structures with a size ranging from 53 to 167 µm (median 124 µm). Those structures were frequently interrupted by pore like structures in the range of 12 to 50 µm. Small pores ranging from 1.0 to 2.5 µm (median 1.3 µm) were also frequently seen (Fig. 1 A, left column). In contrast, BG20 demonstrated a clefty surface with bulgy structures in the size of 6 to 26 µm (median 12.8 µm). The irregular arrangement of those structures resulted frequently in irregularily shaped pore like structures (size range 4–13 µm, median 6 µm) (Fig. 1 A, middle column). Bioglass40 has surface structures in between those of PLA and BG20. The surface is characterized by bulgy alveolar structures which were not as ragged as seen in BG20 and not as smooth compared to PLA. The size of the dominanting surface structure ranged from 24 to 33 µm (median 28.5 µm). Porelike structures were not as frequent in comparison to BG20 (size range 3 to 9 µm, median 6 µm). Interestingly, fiberlike structures were frequently seen (Fig. 1A right column).

The SEM-analysis of sagittal cross sections revealed also significant differences of the inner structures of the biomaterials. PLA demonstrated a relatively regular and homogenous organisation, with large cavities that were connected to pore like structures of the upper surface. BG20 and BG40 were characterized by a significant biphasic structure. The upper side consists of amorphic and clefty structures with cavities and channels which were frequently connected to porelike structures on the surface. Cavities and and pores were frequently smaller in comparison to those observed in PLA scaffolds. The lower side of BG20 and BG40 was much more dense with some small sized pores (Figure 1 B).

The biomechanical analysis revealed a marginally increased ultimate load of scaffolds with bioglass content. The median ultimate load increased slightly with the bioglass content of the scaffold, though significant differences in comparison to PLA were not observed (Figure 1 C).

### Cells

**Figure 2:**
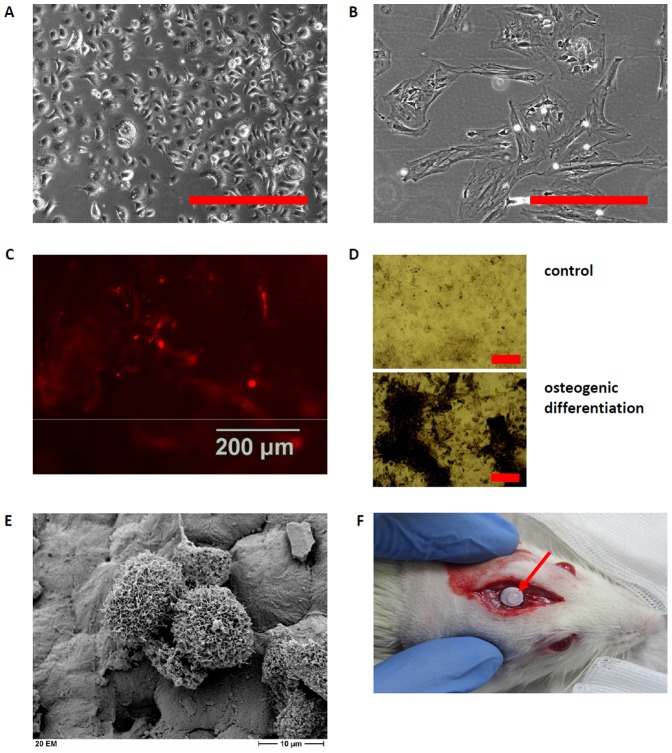
Characteristics of cells *in vitro* and evidence of cells seeded on the scaffold placed in the skull critical size defect. Shape and appearance of EPCs and MSCs obtained from rats in (A) and (B), respectively. Dil-uptake as a marker of endothelial differentiation is exemplarily shown for EPCs seeded on BG20 (C), the calcium deposition as measured by von Kossa staining revealed osteogenic differentiation of MSCs (black areas, D). Adherence of MSCs and EPCs one hour after seeding to the BG20 scaffold (E). MSCs and EPCs can be differentiated by size. Position of scaffold loaded with cells (arrow) in the skull critical size defect of the rat (F). Scale bars: 100 µm (A, B), 200 µm (D), 10 µm (E).

EPCs appeared relatively small with a typical cobblestone like appearance (Fig. 2A). MSCs were larger, flattened and either spindle or irregularly shaped (Fig. 2B). DiL staining demonstrated the endothelial like differentiation of the EPCs (Fig. 2C). Von Kossa staining revealed osteogenic differentiation after incubation with dexamethasone, ascorbic acid and β-glycerolphosphate (Fig. 2D). Cell adherence to the scaffolds was demonstrated by scanning electron microscopy (Fig. 2E). Scaffolds loaded with cells (arrow) were placed in the skull critical size defect of the rat (Fig. 2F).

### Mortality and clinical signs

Neither BG20 and BG40 nor BG20 and BG40, populated with progenitor cells, affected rat mortality. No behavioural changes or visible signs of physical impairment or neurological toxicity were observed during the 3 month observation period. Macroscopic analysis of the implant sites demonstrated comparable scar formation and subsequent healing processes in all four groups. No other test object related clinical signs were observed.

### Histological response at the implantation site

**Figure 3:**
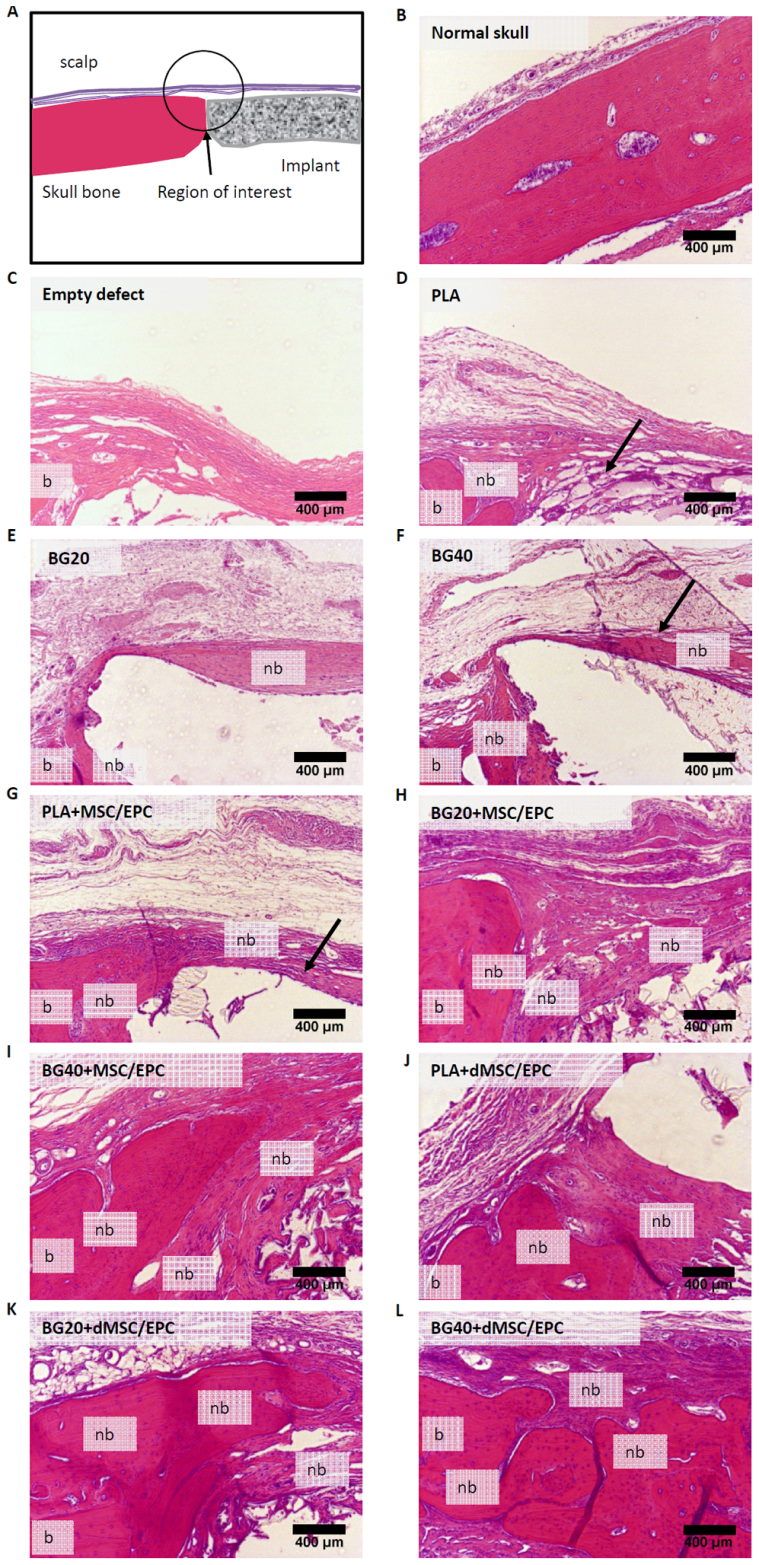
Gross histology of skull defect 3 months after scaffold implantation. Area location (A). Normal rat skull (B). No signs of inflammation are visible. Membrane surrounding the defect (arrows). Staining indicates membrane ossification. Marked increase in new bone formation when implants are populated with progenitor cells and contain a bioglass fraction (G, H, I, J, K, L). PLA = polylactic acid. BG20 = PLA + 20% bioglass, BG40 = PLA + 40% bioglass. MSC = mesenchymal stem cells, dMSC = differentiated mesenchymal stem cells, EPC = endothelial progenitor cells. nb = new bone, b = bone.

The histological response towards the scaffolds is shown in Figure 3. No visible signs of inflammation were noted independently from the scaffolds and cells 3 months after implantation (Fig. 3. B–L). An encapsulation membrane surrounding the implant was frequently visible and most prominent at the upper site of the implant towards the scalp. Various degrees of new bone formation were observed at the contact zone between the skull bone and the implant. Bone formation increased with the percentage of bioglass within the composite implant (Fig. 3 D, E, F). When PLA was implanted, the area of new bone formation was limited to the contact zone of skull and implant (Fig. 3 D). In animals receiving composites with bioglass, bone formation was also directed towards the scalp (Fig. 3 E, F).

A marked increase of bone formation at the contact zone between skull and implant as well as directed towards the scalp was observed in animals receiving implants seeded with progenitor cells. Whether this increase is due to the encapsulation membrane is unsure. The bone formation was more pronounced in animals implanted with BG20 or BG40 with progenitor cells, compared to animals receiving PLA seeded with progenitor cells. (Fig. 3 G, H, I).

The osteogenic predifferentiation of MSCs prior to implantation led to a further increase in bone mass formation. The osteogenic response was more significant if BG20 or BG40 were used as scaffolds, compared to PLA seeded with dMSCs (Fig. 3 J, K , L).

A mild degradation of the margin of cell free implants was observed after 3 months. The degree of degradation and cellular infiltration was more pronounced if BG20 and BG40 were implanted, compared to PLA (Fig. 3 C–F). The addition of progenitor cells led to accelerated cellular infiltration and degradation of the implants. This effect was more pronounced with BG20 and BG40, compared to PLA alone (Fig. 3 G–L).

### Tumorigenicity

**Figure 4:**
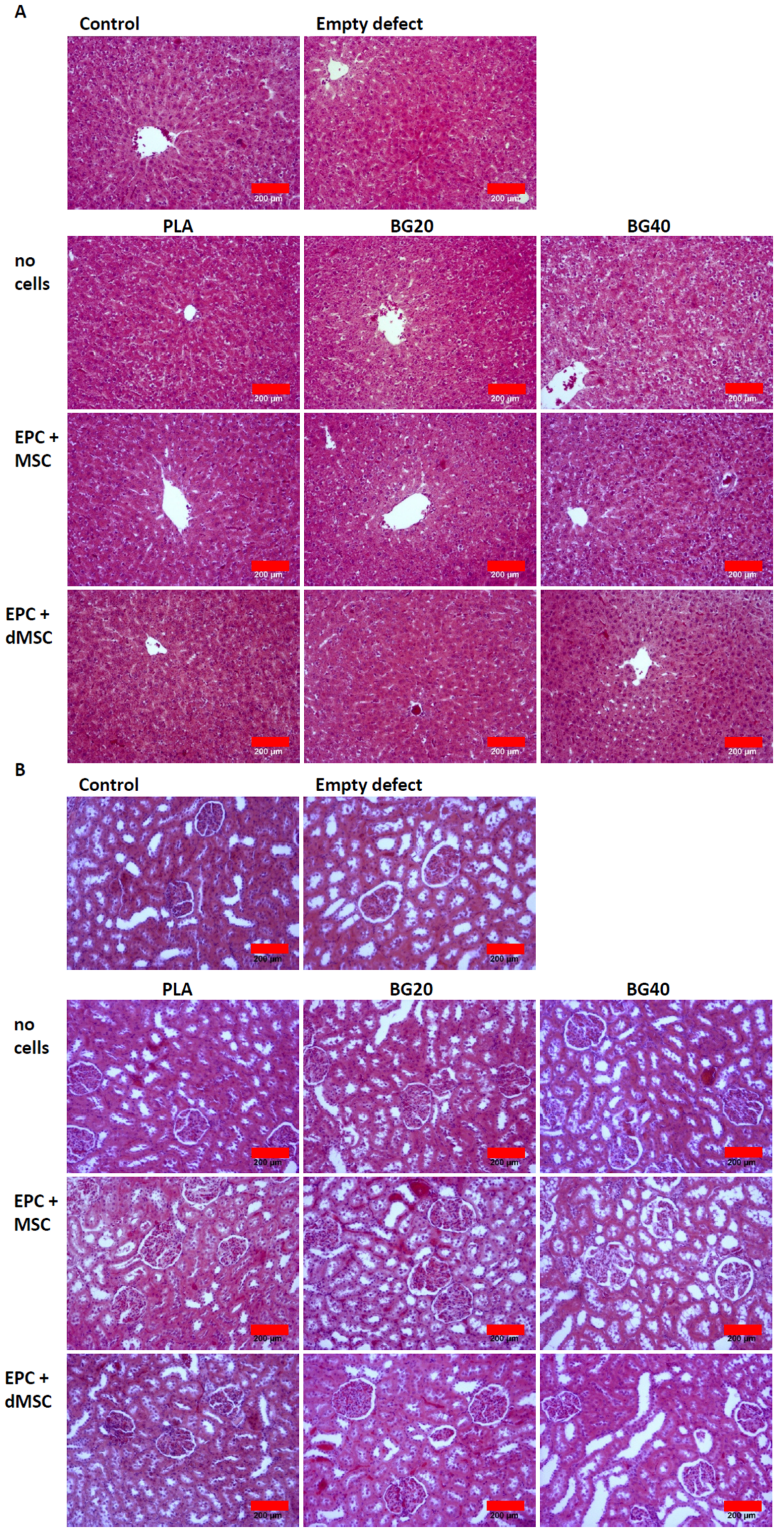
Gross histology of the liver and kidney 3 months after scaffold implantation. No relevant alterations were seen in the treatment groups, compared to control animals and animals with an empty defect. PLA = polylactic acid. BG20 = PLA + 20% bioglass, BG40 = PLA + 40% bioglass. MSC = mesenchymal stem cells, dMSC = differentiated mesenchymal stem cells, EPC = endothelial progenitor cells.

The gross histological analysis of the liver, kidney and spleen revealed no signs of tumor formation (Fig. 4 A, B). Also, no signs of tumor fomation were seen in the macroscopic evaluation of other internal organs (lung, gastrointestinal tract) and the brain during the sacrifice procedure. Accordingly, elevated levels of the surrogate tumor markers MDA and SOD were not observed in the treatment groups, compared to the control group (Table 2).

**Table 2:** Free radical markers after 3 months.

	SOD [unit/mg]	MDA [μMOL/L]	GSH [μMOL/L]	NO_2_ [μMOL/L]
**Empty defect**	0.20 (0.13/0.23)	22.2 (20.5/26.8)	6.5 (4.1/8.1)	15.4 (14.0/16.7)
**PLA**	0.21 (0.2/0.21)	19.9 (11.5/21.3)	4.7 (2.9/5.1)	16.6 (15.1/17.5)
**BG20**	0.22 (0.19/0.22)	24.6 (20.6/26.8)	5.2 (2.6/6.7)	16.4 (14.0/18.2)
**BG40**	0.21 (0.19/0.24)	21.5 (14.8/25.5)	3.7 (3.0/6.0)	16.9 (14.9/20.6)
**PLA+MSC/EPC**	0.20 (0.19/0.21)	15.8* (12.8/17.5)	3.1 (1.9/5.5)	15.2 (14.9/16.9)
**BG20+MSC/EPC**	0.22 (0.20/0.23)	14.0* (12.6/14.8)	4.0 (2.3/4.2)	16.3 (15.5/17.4)
**BG40+MSC/EPC**	0.21 (0.19/0.26)	12.9* (12.5/17.5)	3.7 (2.4/5.2)	16.4 (14.5/18.2)
**PLA+dMSC/EPC**	0.2 (0.19/0.22)	12.8* (12.0/16.0)	4.2 (3.4/5.9)	17.2 (15.3/17.7)
**BG20+dMSC/EPC**	0.2 (0.19/0.21)	11.3* (9.93/12.1)	3.8 (2.7/4.0)	16.7 (15.1/17.4)
**BG40+dMSC/EPC**	0.19 (0.18/0.24)	12.2* (11.0/15.1)	3.8 (2.4/4.9)	16.3 (15.2/18.3)

Results are presented as median (25%-quartile/75%-quartile).

* indicates statistical significance vs. control (empty defect). Additional significant differences are described in the manuscript. SOD = superoxide dismutase, MDA = malondialdehyde, GSH = glutathione. PLA = polylactic acid. BG20 = PLA + 20% bioglass, BG40 = PLA + 40% bioglass. EPC = endothelial progenitor cells, MSC = mesenchymal stem cells, dMSC = osteogenic predifferentiated MSC.

### Body weight and organ status

Weight gain was comparable in the treatment and sham (empty defect) groups. Likewise, the weight of liver, kidney and spleen, normalized to body weight, did not differ among all groups (Table 3). The gross histological analysis of liver and kidney did not reveal any signs of harm or necrosis in any group (Fig. 4 A, B).

**Table 3:** Body weight (bw) and relative organ weight after 3 months.

	Body weight [g]	Liver [% bw]	Kidney [% bw]	Spleen [% bw]
**Empty defect**	563 (528/637)	2.8 (2.7/3.1)	0.63 (0.58/0.71)	0.14 (0.13/0.18)
**PLA**	585 (544/638)	2.9 (2.8/3.1)	0.62 (0.59/0.67)	0.15 (0.14/0.15)
**BG20**	509 (492/574)	2.9 (2.7/3.1)	0.61 (0.55/0.62)	0.13 (0.13/0.15)
**BG40**	575 (540/604)	3.1 (3.0/3.2)	0.58 (0.52/0.65)	0.14 (0.13/0.15)
**PLA+MSC/EPC**	569 (542/640)	3.2 (2.9/3.5)	0.61 (0.55/0.63)	0.14 (0.13/0.15)
**BG20+MSC/EPC**	633 (598/666)	2.9 (2.8/2.9)	0.59 (0.55/0.65)	0.15 (0.13/0.16)
**BG40+MSC/EPC**	615 (569/651)	2.9 (2.6/2.9)	0.59 (0.52/0.65)	0.13 (0.12/0.16)
**PLA+dMSC/EPC**	616 (589/640)	2.7 (2.5/2.8)	0.59 (0.55/0.62)	0.14 (0.13/0.14)
**BG20+dMSC/EPC**	594 (584/650)	2.8 (2.5/3.1)	0.58 (0.56/0.61)	0.14 (0.12/0.15)
**BG40+dMSC/EPC**	605 (587/636)	2.8 (2.6/3.0)	0.61 (0.54/0.64)	0.14 (0.14/0.15)

Results are presented as median (25%-quartile/75%-quartile). PLA = polylactic acid. BG20 = PLA + 20% bioglass, BG40 = PLA + 40% bioglass. EPC = endothelial progenitor cells, MSC = mesenchymal stem cells, dMSC = osteogenic predifferentiated MSC.

### Hematology and inflammation

No relevant differences between the treatment and sham groups were found for any hematological parameter at 3 months. Body temperature of the animals was not significantly different in any group. No significant differences between the groups were apparent for the white blood cell count, whereas the red blood cell count and hemoglobin were slightly decreased in the BG40+dMSC group, when compared to the control group (Table 4).

**Table 4:** Hematologic and inflammatory parameters after 3 months.

	WBC [/nl]	RBC [/pl]	Hemoglobin [g/dl]	Temp. [°C]
**Empty defect**	11.1 (9.8/12.1)	8.8 (8.5/9.3)	15.5 (14.9/15.8)	36 (36/36)
**PLA**	8.9 (7.9/10.4)	8.8 (8.2/9.2)	15.8 (14.8/16.7)	36 (36/36)
**BG20**	10.0 (7.5/11.5)	9.5 (8.8/9.9)	15.7 (15.3/16.2)	36 (36/36)
**BG40**	10.1 (8.4/11.1)	9.9 (8.6/9.9)	16.6 (15.3/17.1)	36 (36/36)
**PLA+MSC/EPC**	9.8 (8.0/13.2)	8.9 (8.7/9.5)	16.3 (15.5/16.5)	36 (36/36)
**BG20+MSC/EPC**	8.8 (8.1/10.0)	8.9 (8.7/9.2)	15.9 (15.0/16.4)	36 (36/36)
**BG40+MSC/EPC**	9.7 (7.2/11.6)	9.1 (8.8/9.5)	16.0 (15.7/16.4)	36 (36/36)
**PLA+dMSC/EPC**	9.8 (8.5/11.3)	8.9 (8.6/9.1)	15.8 (15.4/16.3)	36 (36/36)
**BG20+dMSC/EPC**	7.9 (7.0/8.8)	9.0 (8.5/9.8)	16.7 (16.3/17.6)	36 (36/36)
**BG40+dMSC/EPC**	9.9 (8.0/14.0)	8.4 (7.8/9.1)	15.2 (14.0/16.7)	36 (36/36)

Results are presented as median (25%-quartile/75%-quartile). WBC = white blood cells, RBC = red blood cells. PLA = polylactic acid. BG20 = PLA + 20% bioglass, BG40 = PLA + 40% bioglass. EPC = endothelial progenitor cells, MSC = mesenchymal stem cells, dMSC = osteogenic predifferentiated MSC.

### Serum biochemistry

ALP, GOT, GPT and creatinine were not significantly altered in any group, whereas the serum value of urea was significantly increased in the PLA group, compared to the other groups, with exception of the Sham, BG40 and PLA+dMSC groups. Statistically different values remained within the normal range (Table 5). The serum concentrations of glucose were within the normal range in all groups, however a significant decline was noted for the group BG20+dMSC. Although the total protein content was significantly elevated in the PLA+MSC group in comparison to BG40+dMSC and BG20+MSC, the total protein concentrations were generally similar in all groups (Table 6). Statistically different values remained within the normal range.

**Table 5:** Serum biochemistry (part 1) after 3 months.

	GOT [U/l]	GPT [U/l]	ALP [U/l]	Creatinine [mg/dl]
**Empty defect**	50.0 (28.0/59.8)	20.5 (13.8/25.3)	272 (230/350)	0.40 (0.38/0.43)
**PLA**	41.0 (32.8/49.0)	21.5 (13.8/36.3)	297 (254/399)	0.45 (0.40/0.50)
**BG20**	42.0 (37.0/48.0)	16.0 (13.0/26.0)	253 (179/349)	0.40 (0.40/0.40)
**BG40**	55.0 (33.0/62.0)	22.0 (13.0/29.0)	278 (187/317)	0.40 (0.40/0.50)
**PLA+MSC/EPC**	50.0 (44.0/57.8)	29.0 (14.0/43.0)	263 (220/337)	0.40 (0.40/0.50)
**BG20+MSC/EPC**	28.0 (25.3/39.8)	17.0 (10.8/18.8)	183 (162/207)	0.20 (0.30/0.50)
**BG40+MSC/EPC**	38.5 (26.8/40.0)	21.0 (12.0/26.5)	206 (154/225)	0.40 (0.40/0.48)
**PLA+dMSC/EPC**	33.5 (27.5/41.8)	19.5 (16.8/26.3)	244 (218/300)	0.40 (0.40/0.50)
**BG20+dMSC/EPC**	36.5 (31.5/40.8)	14.0 (11.8/20.8)	211 (180/237)	0.35 (0.30/0.47)
**BG40+dMSC/EPC**	34.5 (27.5/45.3)	18.0 (13.3/25.0)	237 (205/294)	0.40 (0.33/0.40)

Activities of transaminases (GOT/GPT), alkaline phosphatase (ALP) and creatinine is shown. Results are presented as median (25%-quartile/75%-quartile). EPC = endothelial progenitor cells, MSC = mesenchymal stem cells, dMSC = osteogenic predifferentiated MSC.

**Table 6:** Serum glucose, total protein content and urea after 3 months.

	Glucose [mg/dl]	Total protein [g/dl]	urea [mg/dl]
**Empty defect**	325 (285/337)	5.2 (4.6/5.4)	17.0 (15.5/18.5)
**PLA**	299 (223/330)	5.1 (4.9/5.3)	19.5 (17.8/22.0)
**BG20**	318 (277/320)	4.8 (4.4/5.4)	16.0 (15.0/16.0)
**BG40**	286 (271/309)	4.8 (4.6/5.4)	17.0 (14.0/20.0)
**PLA+MSC/EPC**	290 (247/298)	5.8 (5.3/5.9)	13.0 (12.3/14.8)
**BG20+MSC/EPC**	277 (239/311)	4.6 (4.4/4.9)	14.0 (13.0/16.8)
**BG40+MSC/EPC**	246 (229/282)	5.2 (4.7/5.4)	15.0 (14.0/15.8)
**PLA+dMSC/EPC**	256 (221/344)	4.8 (4.8/5.2)	16.0 (15.3/17.8)
**BG20+dMSC/EPC**	213* (207/214)	5.2 (4.6/5.4)	14.0 (13.0/14.8)
**BG40+dMSC/EPC**	261 (234/275)	4.8 (4.5/5.0)	14.0 (14.0/15.8)

Results are presented as median (25%-quartile/75%-quartile).

* indicates statistical significance vs. control (empty defect). Additional significant differences are described in the manuscript. PLA = polylactic acid. BG20 = PLA + 20% bioglass, BG40 = PLA + 40% bioglass. MSC = mesenchymal stem cells, dMSC = osteogenic predifferentiated MSC, EPC = endothelial progenitor cells.

### Free radical markers

Malondialdehyde (MDA), superoxide dismutase (SOD), NO_2_ and glutathione (GSH) were measured as indicators of free radical burden. No significant differences between the treatment group and empty defect group were observed for glutathione, NO_2_ or SOD. Malondialdehyde was significantly increased in the groups “empty defect” and “BG20”, compared to all other groups, with the exception of the “BG40” group. This group had a significantly elevated MDA serum concentration, compared to the groups “PLA+dMSC”, “BG20+dMSC” and “BG40+dMSC” (Table 2).

## Discussion

This study was performed to evaluate the tissue biocompatibility, systemic toxicity and tumorigenicity of a composite material consisting of PLA and bioglass, seeded with various types of progenitor cells in rats over 3 months. The composite materials BG20 and BG40 were well tolerated. Additional regenerative cells seeded on to the scaffold prior to implantation did not evoke adverse reactions such as tumour formation or long-term inflammatory reactions. Histological examination demonstrated that the composite material supports bone formation and bone formation was further enhanced if progenitor cells were seeded onto the biomaterials prior to implantation.

### Characteristics of PLA, BG20 and BG40

The surface structures and perhaps the mechanical stability of disc shaped PLA, BG20 and BG40 were dependent on the bioglass/PLA ratio.

Different surface structures were found on PLA, BG20 and BG40. PLA was rather smooth with very small pores in the range of 1 µm, whereas BG 20 and BG40 offered a rough surface structure with significantly greater pores. Broad evidence exists that the surface characteristic are critical for the ingrowth and function of the cells that were cultivated on a biomaterial [24], [37]. Especially the pore size seems to determine cell's survival. Pore sizes of about 100 µm seem to be ideal [38], [39]. Neither BG20 nor BG40 achieved pores in that size. But besides the pore size, other factors such as surface charge, stiffness, microstructure and the release of bioactive ions are also relevant for the adhesion and survival of the cells [40]. It has been shown that the release of bioactive ions by the biomaterial is highly beneficial for the differentiation and survival of osteoblasts [8], [41], [42], MSC [23] and EPC [43]. Both BG20 and BG40 release Ca-ions which were shown to have a positive effect on survival and differentiation of early EPC [44].

Besides the structural analysis biomechanical issues were also adressed. We observed only a slight, not significant increase of the ultimate load of the disc shaped specimen with increasing bioglass content. Biomechanical properties of composite materials consisting of PLA and bioglass has been also tested by other research groups, though a direct comparison to our disc shaped BG20/BG40 is difficult due to differences in the chemical composition of the bioglass, the size and percentage of bioglass particles and the shape of the specimen. A review of the actual literature revealed inconsistent effects of the bioglass on biomechanical parameters of different composite scaffolds. Zhang and collegues reported about a decrease of tensile strength with increasing percentage of bioglass. In the same study, however, a constant constant tensile strength was observed if the bioglass was pretreated with silane [20]. In contrast Lu and collegues demonstrated a disc shaped composite scaffold made of 50% polylactic-*co*-glycolide (PLGA) and 50% bioglass with higher elastic modulus compared to the PLGA control [21]. Accordingly, Maquet et al reported an improvement of the mechanical properties after the addition of increasing amounts of BG into polymer foams [17].

### Biocompatibility of PLA, BG20 and BG40

No clinical signs of toxicity of the composite materials BG20 and BG40 were apparent. Blood analysis provided no sign for organ damage. GOT, GPT and ALP, all indicators of liver damage, and creatinine and urea, reflecting kidney function, were all within the normal range [45] in all groups. These findings were supported by organ weight analysis and histological examination of the liver and kidney, all of which were without pathological findings in all groups. The lack of visible inflammatory complication at the implantation site, body temperature and white blood cell count provided no signs of a systemic inflammatory reaction 3 months after implantation. Moreover, indicators for free radicals (GSH, NO_2_, SOD) which were associated with inflammation and tumor progression [46] were not increased, compared to the control group.

These findings suggest excellent biocompatibility of BG20 and BG40, which is in line with all available reports in this field. Several scientists have shown that the tissue reaction to PLA is minimal. Among the first, Cutright et al. demonstrated good biocompatibility using pins or a combination of plates and screws for mandibular fixation in dogs. No inflammatory reaction was observed after the initial wound had healed. The implants, however, were surrounded by phagocytic cells [47], [48]. Kang and collegues analyzed the biocompatibility and long-term toxicity of a disc shaped PLA polymer (10 mm diameter) in a critical size calvarial defect in rats in a more recent study. They observed no test material-related effects in mortality, inflammation, hematology, serum biochemistry parameters and organ weight in the operated animals at 8, 12, and 24 weeks after implantation [33]. Other scientists have analyzed the biocompatibility of PLA to cells and consistently report good adhesion and no acute cytotoxic effects on fibroblasts [49], MSCs [50], osteoblasts [51] or endothelial cells [52].

The biocompatibility of silicate based bioglass derivatives has been established. Bioglass was implanted in the maxillae and mandibles of baboons [53], subcutaneously in rats [54] and intramuscularly in rabbits [55]. An adverse inflammatory reaction at the implantation site was not observed in any of these experiments but a thin collagenous capsule can frequently be found at the interface between the bioglass implant and the surrounding tissue [56]. Brandao and colleagues have analyzed the biocompatibility of bioglass implants in the rabbit eviscerated socket. In line with previously published work [53]–[56], no systemic toxicity was observed and a pseudocapsule had formed after 90 days [57].

The compatibility of bioglass towards various cell types *in vitro* was addressed decades ago. Fibroblasts and osteoblasts attach to the bioglass and proliferate normally, lymphocytes and macrophages behave normally, and the phagocytotic activity of macrophages is not depressed [54].

### Biodegradation

Biodegradable biomaterials are of great interest in regenerative medicine, since the implant does not require surgical removal. Minor infiltration and reabsorption of the PLA scaffolds after 3 months was observed in the present study. The slow reabsorption kinetics of PLA implants has also been described by other research groups. Suuronen et al. studied the annual reabsorption of PLA plates in sheep mandibular osteotomies over a period of five years. They observed initial disintegration of the implant after one year and a 52% mass loss of the plates [58]. Cutright et al., with pins or a combination of plates and screws for mandibular fixation in dogs, have described slow reabsorption of PLA-plates, depending on size and shape [47], [48]. Degradation kinetics seem to be strongly dependent on the implantation site. Tschakaloff et al. described a differential loss of PLA-plate mass used for stabilization of nasal bone fractures, implanted in subcutaneous pockets in rabbits. The weight loss of subcutaneously implanted plates was significantly higher after a 42 day observation period [59].

The degradation of bioglass is attributed to solution-mediated dissolution [60]. The dissolution of the implant may allow accelerated in-growth and immigration of cells from tissue close to the implant.

### Tumorigenicity

In terms of systemic toxicology, inflammation and tumorigenicity this investigation has demonstrated the safety of BG20 and BG40. However, the clinical use of in vitro expanded MSCs is still a matter of debate, since they could initiate a tumorigenic process. To reach a sufficient number of MSCs for clinical application it is necessary to expand them. But it has been demonstrated that during *in vitro* expansion chromosomal aberrations and genetic alterations occur, which might lead to malignant transformation [34]. It has been demonstrated that MSCs, co-transplanted with tumor cells, force tumor growth [61]. In contrast, Tarte et al. reported that an accidental transplantation of human aneuploidic MSC occurred without transformation. The patient did not develop any tumors during a two year follow-up [62]. Clinical trials employing MSCs in regenerative medicine have not reported health problems, nor has neoplastic transformation of stem cells been observed at the MSC re-implantation site [63].

In several *in vivo* toxicity studies using NOD/scid mice, rabbits and monkey models it has been shown that the subcutaneous transplantation of culture expanded MSCs does not result in any tumor formation 2 months after injection [64]. EPCs differentiate from monocytic precursors over a five to seven day culture period, and their proliferation potential is not great. They have also been therapeutically applied in humans to treat myocardial infarction. The treatment resulted in significant improvement of the left ventricular ejection fraction and viability of the infarcted area. No adverse effects of the cell therapy were noted at a 4 month follow-up [65]. In this investigation no macroscopically visible signs of tumor formation at the implantation site or in the inner organs were apparent. Histological analysis of the liver, kidney and spleen also yielded no signs of tumor formation. These findings were supported by serum levels of MDA and SOD after 3 months, whereby an increase is associated with tumor progression [46], [66], [67]. Both markers were not significantly increased in the groups receiving the progenitor cells.

Although no tumor formation was observed in the present study it has to be clearly statéd that the number of animals, the observation period and the performed analysis did not allow to exclude a potential risk for tumor formation.

### Bone growth

The qualitative histologic analysis of the defect revealed a higher degree of bone formation if BG20 or BG40 was used. The support of cellular function and angiogenesis by ionic products released by the bioglass has been addressed in recent studies [43]. This effect might support the in-growth of cells into the implant and in this investigation EPC and MSC seeding led to a further increase in new bone mass. In a previous study it was also possible to demonstrate that the combination of EPCs and MSCs lead to significantly increased bone mass, compared to EPC or MSC application alone. This bone mass increase may be attributable to significantly improved EPC induced early vascularization. Enhanced early vascularization enhances nutritional support in the defect site, improving MSC survival and function [30]–[32].

### Future implications and conclusion

Evidence is provided here for excellent biocompatibility of the biomaterials BG20 and BG40, moreover the biomaterials BG20 and BG40 seeded with progenitor cells supported the formation of new bone mass in a calvarial defect rat model. Though, it might be of interest to what extend cellular adherence, growth and bone healing response will be influenced by the shape of those biomaterials. Moreover, the suitability of BG20 and BG40 for other kinds of bone defect has to be proven in further experimental studies. Additionally, further studies are required to adress the risk of tumorgenicity of those biomaterials in combination with regenrative cells sufficiently.
